# *Paenibacillus*–*Pseudomonas* Consortium Improves Barley Performance with Minimal Impact on Native Rhizobacterial Community

**DOI:** 10.3390/microorganisms14020488

**Published:** 2026-02-18

**Authors:** Jakub Dobrzyński, Aleksandra Naziębło, Iryna Kulkova, Magdalena Szpytma, Adrianna Antosik, Monika Sitarek-Andrzejczyk, Barbara Wróbel

**Affiliations:** 1 Institute of Technology and Life Sciences-National Research Institute, Falenty, 3 Hrabska Avenue, 05-090 Raszyn, Poland; a.nazieblo@itp.edu.pl (A.N.); i.kulkova@itp.edu.pl (I.K.); m.szpytma@itp.edu.pl (M.S.); m.sitarek-andrzejczyk@itp.edu.pl (M.S.-A.); b.wrobel@itp.edu.pl (B.W.); 2 Ekosystem-Nature’s Heritage Association, Institute of Microbial Technologies, Al. NSZZ Solidarność 9, 62-700 Turek, Poland; adrianna.antosik@itm.turek.pl

**Keywords:** plant-growth promoting bacteria, native rhizobacteria, barley, microbial ecology, 16S rRNA gene sequencing

## Abstract

The intensive use of mineral nitrogen fertilizers in cereal production contributes to environmental degradation, highlighting the need for more sustainable crop management strategies. Plant growth-promoting bacteria (PGPB) offer a promising alternative; however, their effects on native rhizosphere communities remain underexplored, particularly in barley. This study evaluates the impact of a bacterial consortium composed of *Paenibacillus* sp. Z15 and *Pseudomonas* sp. KR227 on barley growth, yield, and rhizosphere bacteria under field conditions in temperate climate (2025). Plant biometric traits, photosynthetic pigment content, and soil properties were measured, and rhizobacterial communities were analyzed using 16S rRNA gene (V3–V4) sequencing. The PGPB consortium significantly increased early root biomass (120%), shoot height (7.8%), and grain yield (15.5%), while no significant effects were observed on soil chemistry or photosynthetic pigments. Sequencing revealed no major changes in alpha or beta diversity; however, transient shifts in the relative abundance of specific taxa were detected relatively shortly after inoculation and mostly disappeared by harvest. These findings indicate that the *Paenibacillus–Pseudomonas* consortium can enhance barley performance without disrupting native rhizobacterial communities. Overall, the results support the potential of PGPB as a sustainable agronomic tool and provide new insights into PGPB–microbiome interactions in barley under field conditions.

## 1. Introduction

Barley (*Hordeum vulgare* L.) is one of the oldest and most economically important cereal grains. Globally, it ranks fifth in organic matter production, following maize, wheat, rice, and soybean, and preceding crops such as sugarcane, potato, and sorghum [1]. Although barley was likely first cultivated as a food source for humans, its use has progressively shifted toward animal feed, malting, and brewing, partly due to the increasing dominance of wheat and rice in human diets. Barley is considered the most adaptable cereal grain, as it can thrive across a broader range of environmental conditions than other cereals. It is cultivated at higher latitudes, greater altitudes, and in drier regions than most other cereal species [2].

Like other crops, barley requires substantial inputs of nitrogen (N) and phosphorus (P) fertilizers to achieve high yields. However, the intensive use of mineral fertilizers contributes to environmental pollution [3]. Nitrogen fertilization can result in nitrate leaching into groundwater and emissions of nitrous oxide (a potent greenhouse gas), whereas phosphorus fertilizers may cause soil accumulation and runoff, leading to the eutrophication of surface waters [4]. In response to these environmental challenges, the European Union has introduced several initiatives, including the EU Soil Strategy for 2030 and the Nitrates Directive, aimed at reducing nitrogen and phosphorus inputs in order to protect soil and water resources [5].

One promising approach to achieve these objectives is the use of plant growth-promoting bacteria (PGPB). These beneficial microorganisms can improve nutrient availability, stimulate plant development, and ultimately reduce reliance on synthetic fertilizers, thereby offering a more sustainable strategy for crop production [6,7]. PGPB may promote plant growth through direct mechanisms, such as solubilizing phosphate, fixing atmospheric nitrogen, or producing phytohormones that stimulate plant development [8,9,10,11]. In addition, they can enhance plant performance indirectly by mitigating biotic and abiotic plant stresses through the production of antibiotic compounds, the synthesis of hydrolytic enzymes, or by triggering induced systemic resistance (ISR) [12,13,14].

To date, numerous studies have examined the effects of PGPB on barley growth [15,16,17,18,19]; however, only a limited number have investigated their impact on the native microbiota, particularly the root-associated bacterial community [20]. Moreover, to our knowledge, no previous studies have assessed these interactions along the continuum between PGPB and the native rhizobacterial community of barley using high-resolution sequencing methods that provide relatively fine taxonomic resolution. Therefore, the objective of the present study was not only to evaluate the effects of our plant growth-promoting bacterial inoculants on barley growth and yield, but also to determine their influence on the diversity and taxonomic composition of the native barley rhizosphere microbiota using Next-Generation Sequencing (NGS).

## 2. Materials and Methods

### 2.1. Bacterial Strains Isolation, Selection and Identification

The study aimed to investigate bacterial nitrogen fixation and phosphate solubilization. Accordingly, the consortium was composed of strains exhibiting these functional traits, selected from a pool of approximately 100 isolates obtained from grassland ecosystems. More detailed information on the initial screening and selection procedures has been reported in our previous studies [21,22]; however, a brief description is provided below for clarity.

The bacterial consortium consisted of *Pseudomonas* sp. KR227 and *Paenibacillus* sp. Z15. *Pseudomonas* sp. KR227, isolated from bulk grassland soil, has been characterized in our previous research [21] and exhibits the ability to solubilize phosphate and produce indole-3-acetic acid (IAA), a phytohormone of the auxin class [21]. Strain *Paenibacillus* sp. Z15 was isolated from the rhizosphere of perennial ryegrass (*Lolium perenne* L.) and has also been described in our previous research [22]. Based on classical as well as molecular techniques, the strain was classified as a diazotrophic bacterium [22].

Taxonomic identification of isolate Z15 was conducted using standard procedures based on 16S rRNA gene sequencing, as described previously [21,22]. To improve taxonomic resolution, the housekeeping gene *gyrB*, involved in basic metabolic processes, was also analyzed. Genomic DNA from a 16 h culture of the isolate was amplified using the degenerate primers UP-1 (forward, 5′-CAYGCNGGNGGNAARTTYGA-3′) and UP-2r (reverse, 5′-TCNACRTCNGCRTCNGTCAT-3′), following [23]. PCR conditions were as follows: initial denaturation at 95 °C for 3 min; 35 cycles of denaturation at 95 °C for 30 s, annealing at 55 °C for 45 s, and extension at 72 °C for 90 s; and a final extension at 72 °C for 5 min. Purified PCR products were sequenced by the Sanger method (NEXBIO, Lublin, Poland).

The obtained 16S rRNA and *gyrB* gene sequences of Z15 were compared with the GenBank database using Basic Local Alignment Search Tool, (BLAST, version: +2.17.0). In addition, a phylogenetic tree was created using MEGA 12 software. Marker gene sequences of *P. polymyxa* and *P. peoriae* type strains, together with the highest scoring BLAST sequences, were retrieved from GenBank. Sequences were aligned separately for each gene using MUSCLE implemented in MEGA 12. The alignments were subsequently concatenated, and a phylogenetic tree was constructed using the Maximum Likelihood method based on the Tamura–Nei model with 1000 bootstrap replicates.

### 2.2. Field Experiment, Soil Sampling, and Plants Measurement

The field experiment was conducted at the experimental farm of the Institute of Technology and Life Sciences–National Research Institute in Falenty, Mazowieckie Voivodeship, Poland (52.136256, 20.918258). Spring barley (*Hordeum vulgare* L.; Farmer variety) was sown in April 2025 at a seeding rate of 400 grains m^−2^. The soil was classified as Luvisols according to the World Reference Base for Soil Resources (WRB), and maize was the preceding crop. Prior to sowing, mineral N fertilization was applied at a rate of 70 kg N ha^−1^ in the form of urea (46% N), with no additional nitrogen fertilization applied during the growing season. At the onset of the experiment, the average soil pH was approximately 6.5.

Meteorological conditions during the experimental period (April–July 2025) were as follows: in April, the mean air temperature was 11.8 °C and precipitation totaled 30.1 mm; in May, the mean temperature was 12.0 °C with 72.6 mm of rainfall; June was characterized by a mean temperature of 18.5 °C and 47.7 mm of precipitation; and in July, the mean temperature reached 20.0 °C with 53.8 mm of precipitation.

The experiment was established in late April 2025 on a production field using a randomized complete block design with four replicates. Individual plots measured 10 m^2^ (1.5 m × 6.7 m) and were arranged as narrow, elongated, separated by 1 m buffer zones. Two treatments were applied: a non-inoculated control (C) and a bacterial consortium (PP) composed of *Paenibacillus* sp. Z15 and *Pseudomonas* sp. KR227. Inoculation was carried out in early May 2025 at the early tillering stage of barley (BBCH 21). The bacterial strains were cultured separately in lysogeny broth (LB) for 24 h. Each culture was adjusted to the target cell density using a densitometer and was previously validated by serial dilution and plating method on nutrient agar. The strains were then mixed in equal proportions (1:1). The resulting bacterial suspension, containing 1.5 × 10^8^ cells mL^−1^ in sterile saline solution (0.9%), was applied with a hand-held sprayer positioned approximately 40 cm above the soil surface, delivering 600 mL of inoculum per plot.

Rhizosphere soil was sampled twice during the growing season: three weeks after inoculation (I term-BBCH 30) and at crop harvest (II term). At each sampling date, roots from nine plants per plot were excavated, and three composite samples were prepared per treatment. Bulk soil loosely adhering to the roots was removed by gentle shaking, while the soil tightly attached to the root surface was collected using a sterile brush and passed through a sterile sieve, following established protocols [24,25]. Soil samples were stored at −20 °C for chemical analyses and at −80 °C for molecular analyses.

Three weeks after inoculation, root biomass was determined for ten plants per plot at BBCH 30. Subsequently, leaves were sampled from plants at BBCH 45. At harvest, plants from a 1 m^2^ area within each plot were sampled to determine plant height (20 plants), root biomass (20 plants), shoot yield, grain yield, and thousand grain mass (TGM).

### 2.3. Physico-Chemical Properties

Soil physico-chemical analyses were conducted in accordance with ISO and PN-EN standards. Soil pH (measured in KCl) was determined following PN-ISO 10390:2022-09 [26]. Total carbon (TC) content was measured according to PN-ISO 15934:2012 [27]. The nitrogen content (TKN) was determined using the Kjeldahl method in accordance with PN-EN 16169:2012 [28]. Mineral forms of nitrogen, including ammonium (N-NH_4_) and nitrate (N-NO_3_), were quantified using Spectroquant^®^ test kits (Merck, Germany). Available phosphorus (AP) and total phosphorus (TP) were measured using methods based on Mehlich 3 extraction and ICP-OES detection, following PN-EN 54321:2021-07 [29] and PN-EN ISO 22036:2024-07 [30] standards.

### 2.4. Photosynthetic Pigments

Chlorophyll *a*, chlorophyll *b*, and total carotenoid contents were quantified in fresh leaf tissues using a modified method based on Arnon [31] and Azeem et al. [32]. Briefly, 0.5 g of fresh leaf material was homogenized in 10 mL of 80% (*v*/*v*) acetone. The homogenate was kept in the dark at 4 °C for 24 h and subsequently centrifuged. The absorbance of the resulting supernatant was measured at 480, 645, and 663 nm using a UV–Vis spectrophotometer.

Pigment concentrations were calculated using the following equations:

Chl*a* = [12.7 (A663) − 2.69 (A645)] × V/1000 × W,

Chl*b* = [22.9 (A663) − 4.68 (A645)] × V/1000 × W,

Car = [(A480) − 0.114 (A663) − 0.638 (A645)] × 1000/2500,

where

Chl*a*/*b*—chlorophyll *a*/*b* content [mg/g FW];

Car—carotenoid content [mg/g FW];

A—absorbance at different wavelengths;

V—solvent volume [ml];

W—weight of the tissue [g].

### 2.5. 16S rRNA Sequencing and Bioinformatic Analyses

Soil DNA was extracted using the Magnetic Soil and Stool DNA Kit (Tiangen, China). The V3–V4 region of the 16S rRNA gene was amplified by PCR using primers 314F (5′-CCTACGGNGGCWGCAG-3′) and 785R (5′-GACTACHVGGTATCTAATCC-3′). Each PCR mixture contained 15 μL of Phusion^®^ High-Fidelity PCR Master Mix, 0.2 μM of each primer, and approximately 10 ng of template DNA. PCR cycling conditions consisted of an initial denaturation at 98 °C for 1 min, followed by 30 cycles of denaturation at 98 °C for 10 s, annealing at 50 °C for 30 s, and extension at 72 °C for 30 s, with a final extension at 72 °C for 5 min. PCR products of the expected size were verified by electrophoresis on 2% agarose gel.

Sequencing libraries were prepared using the NEB Next^®^ Ultra™ II FS DNA PCR-free Library Prep Kit (New England Biolabs, Ipswich, MA, USA) following the manufacturer’s instructions. Sequencing was performed on an Illumina NovaSeq 6000 platform (Novogene, Planegg, Germany) in paired-end mode, producing 250 bp reads. Raw sequences were quality filtered and de-duplicated using the DADA2 approach, and unique sequences were defined as Amplicon Sequence Variants (ASVs). Taxonomic assignment was performed using a Naive Bayes classifier against the Silva 138.1 database. Based on taxonomic profiles, the ten most abundant phyla and genera were selected for the presentation of relative abundances.

Alpha diversity was calculated in QIIME2 using Observed_features, Chao1, Shannon, and Simpson indices. Beta diversity was assessed by principal coordinate analysis (PCoA) based on Bray–Curtis dissimilarity using the ade4 and ggplot2 packages in R (version 4.0.3). Differences in bacterial community composition were tested using Analysis of Similarities (ANOSIM) with the vegan package (version 2.5-7) in R (version 4.0.3). Functional profiling of the rhizobacterial community was inferred using PICRUSt, a bioinformatic approach that predicts metagenomic functional potential based on 16S rRNA gene sequences. The inferred functional pathways were subsequently annotated and classified using the Kyoto Encyclopedia of Genes and Genomes (KEGG) database.

### 2.6. Statistical Analysis

LEfSe and *t*-test analyses were used to identify significant differences in bacterial community composition at the phylum and genus levels using the vegan and ggplot2 packages in R. Differences in soil chemical parameters, photosynthetic pigment contents, and biometric traits were evaluated using Tukey’s HSD test (*p* < 0.05). Tukey’s HSD tests (one-way ANOVA) were performed using Statistica 13.1 [33].

## 3. Results

### 3.1. Bacterial Strain Identification

As mentioned above, based on the plate assay conducted on nitrogen-free medium and the detection of the *nifH* gene, strain Z15 was identified as a diazotrophic bacterium, in agreement with previous reports [22]. Taxonomic identification in earlier work included an analysis of the 16S rRNA gene sequence followed by a BLAST comparison, which assigned strain Z15 (accession number PX381315, GenBank) to the genus *Paenibacillus* [22]. In the present study, an additional housekeeping gene, *gyrB*, was sequenced (Supplementary Materials; Figure S1), and a phylogenetic tree was constructed based on the concatenated 16S rRNA and *gyrB* sequences. Isolate Z15 formed a well-supported clade with *P. polymyxa* NK-1 but did not cluster with the type strains of either *P. polymyxa* or *P. peoriae*. This multilocus approach confirmed the placement of strain Z15 within the genus *Paenibacillus;* however, it did not enable an unambiguous assignment at the species level. The phylogenetic tree is provided in the Supplementary Materials (Figure S1).

### 3.2. Physico-Chemical Properties of Soil

Application of the PP consortium did not result in statistically significant changes in soil properties relative to the control. Moreover, no significant differences were observed either three weeks after inoculation (term I) or at harvest (term II) (Table 1). Total organic carbon (TOC) ranged from 0.78% to 0.98%, while total Kjeldahl nitrogen (TKN) ranged from 0.11% to 0.12% of the sample mass. The barley rhizosphere contained 5.4–5.67 mg/kg ammonium nitrogen (N-NH_4_) and 2.25–4.73 nitrate nitrogen (N-NO_3_), as well as 224–277.33 mg/kg available phosphorus (AV) and 539–629.33 mg/kg total phosphorus (TV). Soil pH values varied between 6.17 and 7.00.

### 3.3. Photosynthetic Pigments Content

**Figure 1:**
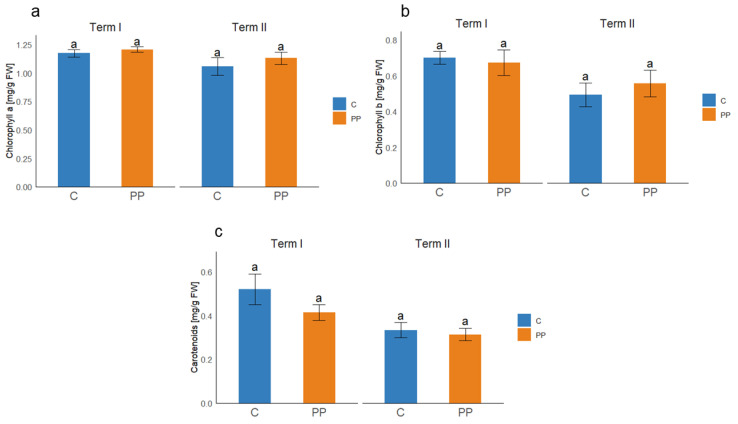
Effect of PP consortium inoculation on (**a**) chlorophyll *a*, (**b**) chlorophyll *b*, and (**c**) carotenoids content in fresh plant material; C—control, PP—inoculation with the consortium. The graphs present mean values ± standard deviations. Different letters in the same sampling term indicate significant differences between means at *p* < 0.05 according to Tukey’s HSD test.

Inoculation with the PP consortium did not result in statistically significant changes in the content of photosynthetic pigments compared with the control at either sampling term (Figure 1). Chlorophyll *a* concentration remained stable across treatments and terms, ranging from 1.06 to 1.17 mg/g FM in control plants and from 1.13 to 1.21 mg/g FM in PP-treated plants. At the first sampling date, chlorophyll *b* levels were 0.7 and 0.67 mg/g FM in the control and PP-treated plants, respectively, whereas at the second sampling they were 0.49 and 0.56 mg/g FM. Similarly, carotenoid contents were lower at term II in both groups (0.33 and 0.31 mg/g FM) compared to term I (0.52 and 0.41 mg/g FM).

### 3.4. Biometric and Yield Parameters of Barley Plants

Application of the PP consortium had a significant effect on several biometric traits of barley plants (Table 2). Shoot height was significantly greater in inoculated plants, increasing by 7.78% relative to the control. Grain yield was also significantly enhanced by 15.5% in the PP treatment compared with the control. Root mass measured three weeks after inoculation (first sampling date) was significantly higher in PP-treated plants, with a 120% increase relative to the control. In contrast, root mass at harvest (second sampling date) did not differ statistically between treatments. Thousand grain weight (TGW) remained relatively constant across treatments, ranging from 4.74 to 4.79 g, and did not show a clear response to inoculation. Similarly, shoot yield did not differ significantly between treatments, with mean values of 1233.75 g for PP-treated plants and 1049 g for control plants.

### 3.5. Alpha Diversity and Beta Diversity of Bacterial Community

No statistically significant differences in the alpha diversity of rhizosphere-associated bacteria were detected between treatments. The alpha diversity of barley rhizobacterial communities was evaluated using Chao1, observed features, and the Shannon and Simpson indices (Table 3). At the first sampling date, control samples exhibited a Chao1 value of 2484 and 2457 observed features, whereas PP-inoculated samples showed slightly higher values of 2510 and 2485, respectively. Shannon diversity was comparable between treatments (10.34 in the control and 10.33 in PP-treated samples), and the Simpson index remained consistently high—0.998 in both treatments. At the second sampling date, alpha diversity decreased slightly in both treatments. Control samples exhibited a Chao1 of 1799 and 1778 observed features, while PP-treated samples showed values of 1946 and 1926, respectively. Shannon index ranged from 9.88 in the control to 9.94 in the PP treatment, and the Simpson index remained stable at 0.998 across treatments.

**Figure 2:**
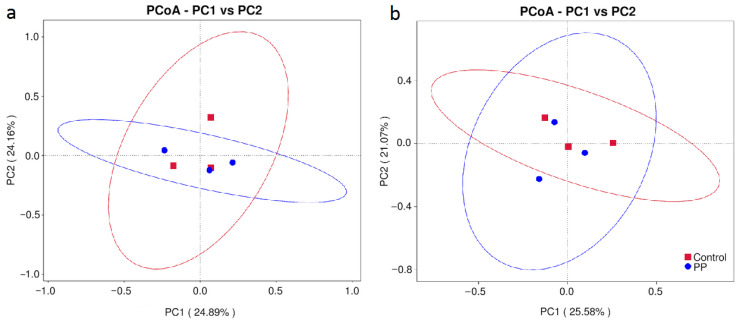
PCoA of rhizosphere bacterial communities in barley based on Bray–Curtis dissimilarity; three weeks after inoculation—term I (**a**) and at harvest—term II (**b**); PP—inoculation with bacterial consortium. Ellipses represent 95% confidence intervals for each treatment group: Control—red Control, PP—blue.

Principal Coordinates Analysis (PCoA) based on Bray–Curtis dissimilarity was used to assess the beta diversity of rhizosphere bacterial communities in barley (Figure 2). At the first sampling date, PC1 and PC2 explained 24.89% and 21.16% of the total variation, respectively, and no clear separation between treatments was observed. Similarly, at the second sampling date, PC1 and PC2 accounted for 25.58% and 21.07% of the variation, and the overall community structure remained largely overlapping between PP and the control. ANOSIM analysis confirmed the absence of significant differences in community composition between treatments (first term: R = −0.11111, *p* = 0.7; second term: R = −0.22222, *p* = 0.9).

### 3.6. Composition of Bacterial Community

**Figure 3:**
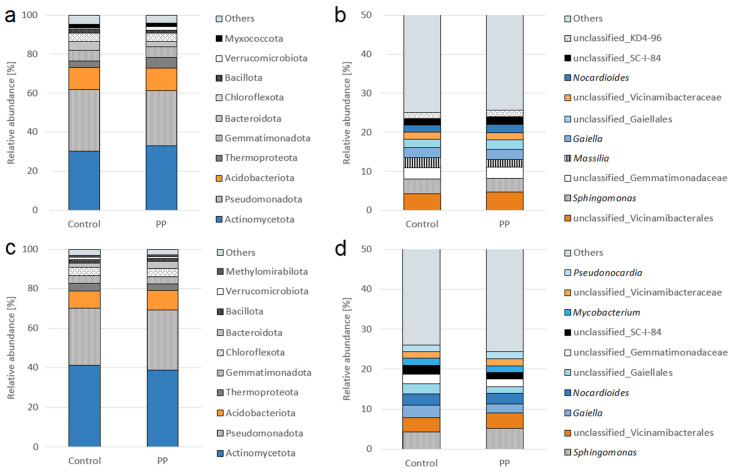
Relative abundance of predominant bacterial phyla (**a**,**c**) and genera (**b**,**d**) in the barley rhizosphere, three weeks after inoculation—term I (**a**,**b**) and at harvest—term II (**c**,**d**); PP—inoculation with bacterial consortium.

At the first sampling term, the dominant bacterial phyla were Actinomycetota (30.40–33.11%), Pseudomonadota (28.06–31.37%), and Acidobacteriota (11.48–11.57%). Other relatively abundant phyla included Thermoproteota (3.33–5.48%) and Gemmatimonadota (5.22–5.42%). Bacteroidota accounted for 3.01–4.73% of the community, while Chloroflexota represented approximately 4.15–4.17%. Less abundant phyla included Bacillota (1.61–2.20%), Verrucomicrobiota (0.62–1.74%), and Myxococcota (1.64–1.70%). Taxa grouped as “Others” comprised 4.22–4.76% of the total community (Figure 3a). At the second sampling term, Actinomycetota (38.89–41.35%), Pseudomonadota (28.88–30.36%), and Acidobacteriota (8.71–9.76%) remained the dominant bacterial phyla. These were followed by Chloroflexota (4.02–4.19), Gemmatimonadota (3.76–4.05%), and Thermoproteota (3.35–3.75%). Lower relative abundances were observed for Bacteroidota (2.35–3.53%), Bacillota (1.45–1.70%), Verrucomicrobiota (0.85–1.11%), and Methylomirabilota (0.91–0.99%). Taxa classified as “Others” accounted for 4.22–4.76% of the community (Figure 3c).

At the genus level, the most abundant taxa at the first sampling term were unclassified_Vicinamibacterales (4.33–4.72%) and *Sphingomonas* (3.49–3.74%). ASVs assigned to unclassified_Gemmatimonadaceae accounted for 2.81–2.83% of the community, while *Massilia* represented 2.01–2.65%. *Gaiella* showed a relative abundance of 2.51–2.58% and unclassified_Gaiellales accounted for 2.13–2.43%. Similar proportions were observed for unclassified_Vicinamibacteraceae (1.88–2.02%) and *Nocardioides* (1.82–2.01%). Among the least abundant of the listed taxa were unclassified_SC-I-84 (1.65–2.03%) and unclassified KD4-96 (1.55–1.6%). Low-abundance taxa grouped as “Others” constituted the largest fraction of the community, representing approximately 74–75% (Figure 3b). At the second sampling date, the predominant taxa were *Sphingomonas* (4.38–5.24%), unclassified_Vicinamibacterales (3.56–3.88%), *Gaiella* (2.11–3.01%), and *Nocardioides* (2.77–2.87%). Other relatively abundant taxa included unclassified_Gaiellales (1.59–2.51%), unclassified_Gemmatimonadaceae (1.97–2.40%), unclassified_SC-I-84 (1.60–2.20%), *Mycobacterium* (1.61–1.83%), unclassified_Vicinamibacteraceae (1.60–1.89%), and *Pseudonocardia* (1.67%). Taxa classified as “Others” accounted for 73.97–75.68% of the community (Figure 3d).

**Figure 4:**
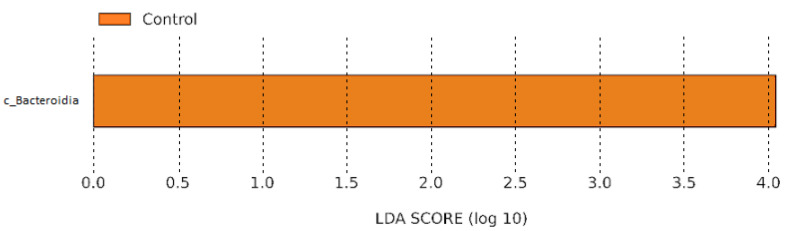
LEfSe analysis of the barley rhizobacterial community three weeks after inoculation (term I) with bacterial consortium PP.

Changes in bacterial community in response to PP consortium application affected only a small fraction of the predominant taxa. At the first sampling term, LEfSe analysis revealed a significantly higher relative abundance of members of the class Bacteroidia in the control treatment, with an LDA score (log 10) exceeding 4.0, indicating that this group was a key contributor to the observed differences between treatments. However, no significant differences were detected between treatments at the second sampling term (Figure 4).

**Figure 5:**
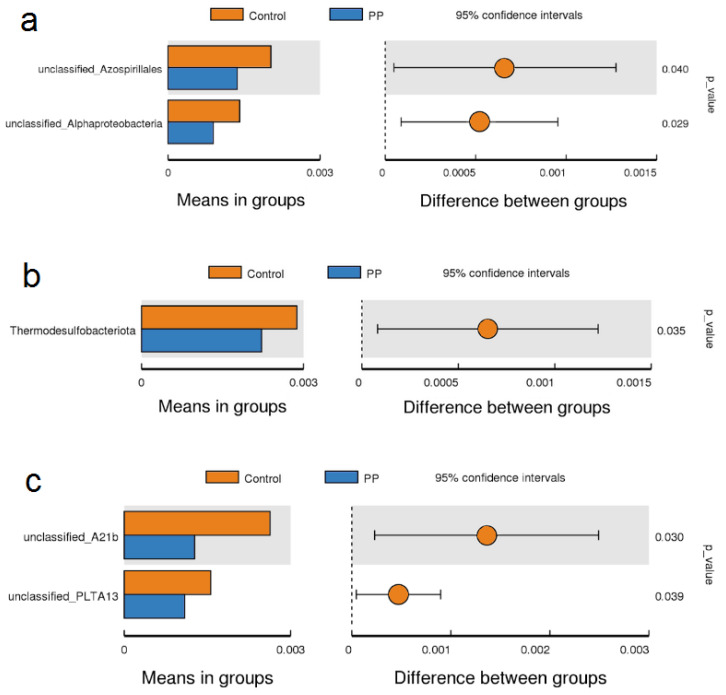
*t*-test of the barley rhizobacterial community of barley (a,c—genera, b—phyla), three weeks after inoculation—term I (**a**,**b**) and at harvest—term II (**c**). PP—inoculation with bacterial consortium. The graphs display the 95% confidence intervals for the microbial groups.

In contrast to the LEfSe results, the *t*-test identified differentially abundant taxa, indicating that the effects of PP consortium inoculation on specific bacterial groups were subtle and varied between sampling times. At the first sampling date, *t*-test analysis revealed a significantly lower relative abundance of unclassified Azospirillales (Alphaproteobacteria) and unclassified Alphaproteobacteria in the PP-inoculated rhizosphere compared with the control (Figure 5a). In addition, Thermodesulfobacteriota exhibited a significantly reduced relative abundance in the PP treatment at this time point (Figure 5b). At the second sampling date, a significantly lower relative abundance of unclassified A21b (Betaproteobacteria) and unclassified PLTA13 (Gammaproteobacteria) was observed in the inoculated rhizosphere relative to the control (Figure 5c).

Furthermore, PICRUSt-based analysis of 16S rRNA data (Supplementary Materials; Figure S2) revealed no significant differences (*t*-test) in the inferred functional potential of rhizobacterial communities following at either the first or the second sampling time point.

## 4. Discussion

Bacteria belonging to the genera *Paenibacillus* and *Pseudomonas* are among the most extensively studied PGPB; however, to date, relatively few studies have specifically examined their potential to enhance barley growth under field conditions [16,18,19].

In the present field experiment, application of a consortium composed of *Paenibacillus* and *Pseudomonas* strains did not result in statistically significant changes in the chemical properties of the barley rhizosphere soil. These findings are not fully consistent with some reports in the literature, in which inoculation with phosphate-solubilizing bacteria led to increased concentrations of available phosphorus, or inoculation with nitrogen-fixing bacteria resulted in higher ammonium nitrogen contents [21,34]. In the present study, however, it is plausible that plants rapidly utilized the available nutrient forms, thereby limiting their detectability at the specific time points. In addition, the apparent discrepancy between plant growth stimulation and nutrient availability in soil may reflect methodological limitations associated with the determination of nitrogen and phosphorus concentrations in soil [35].

Despite the absence of detectable changes in soil chemical properties, a significant increase in root biomass was observed three weeks after inoculation (term I), and by the end of the experiment, both shoot length and grain yield were enhanced in response to the bacterial consortium. The observed stimulation of plant growth can likely be attributed to multiple complementary mechanisms, including IAA production by *Pseudomonas* sp. KR227, as well as its phosphate-solubilizing capabilities and the nitrogen-fixing potential of *Paenibacillus* sp. Z15. On the other hand, PGPB application did not result in higher chlorophyll *a* content, either three weeks after inoculation (BBCH 30) or at later stages of plant development closer to the end of the growing period (BBCH 45).

Comparable effects of PGPB on barley growth have been reported by other authors. Under controlled growth conditions, Kaur et al. [18] demonstrated that soil inoculation with PGPB applied either individually or as a microbial consortium significantly enhanced barley growth and physiological parameters relative to untreated control. In their study, a consortium composed of the nitrogen-fixing *Erwinia* sp. EU-B2SNL1, the phosphorus-solubilizing *Chryseobacterium arthrosphaerae* EU-LWNA-37, and the potassium-solubilizing *Pseudomonas gessardii* EU-MRK-19 was particularly effective, promoting root length, shoot height, biomass accumulation, and increasing leaf chlorophyll and carotenoid contents [18]. Similarly, under field conditions, seed inoculation of barley with N-fixing strains *Azotobacter chroococcum* (Azt) and *Azospirillum lipoferum* (Azs) positively influenced plant growth [17]. The combined application of both strains resulted in increased plant height, grain yield, and yield components, as well as elevated leaf chlorophyll and carotenoid contents and improved plant water status [17].

To date, only a limited number of studies have investigated the effects of PGPB, including members of the genera *Paenibacillus* and *Pseudomonas*, on the soil microbiome associated with barley. Previous research has primarily focused on changes in microbial activity [15,36] or on overall barley rhizosphere diversity assessed using traditional, low-resolution methods [20]. To the best of our knowledge, the present study is the first to examine interactions between PGPB inoculants and the native bacterial community of the barley rhizosphere using next-generation sequencing techniques.

Our results demonstrate that application of the PP consortium did not induce significant changes in either alpha or beta diversity. Similar observations have been reported by other authors following the application of various PGPB (including rhizobacteria) to different plant species [37,38]. Nevertheless, three weeks after the start of the experiment, LEfSe analysis revealed a decrease in the relative abundance of taxa belonging to the class Bacteroidia in the rhizosphere of PP-treated plants. The ecological implications of this shift remain difficult to interpret, as the class Bacteroidia is less extensively characterized with respect to its functional roles in soil compared with other classes within the phylum Bacteroidota, such as Flavobacteriia and Sphingobacteriia [39,40]. Importantly, this shift was no longer detectable at harvest, indicating that the observed effect on Bacteroidia abundance was transient in nature.

In addition, *t*-test analysis revealed further shifts in specific components of the native barley rhizosphere. Three weeks after inoculation, a decrease in unclassified Azospirillales (Alphaproteobacteria) and a general reduction in unclassified Alphaproteobacteria were observed. Given that the class Alphaproteobacteria includes well-known nitrogen-fixing genera such as *Azospirillum*, which commonly occurs in soil [41,42], such a shift could be considered potentially unfavorable. However, no significant negative changes were detected at the genus *Azospirillum* level in PP-inoculated samples. At the second sampling point, decreases were observed in other lineages within the phylum Pseudomonadota, specifically unclassified A21b (Betaproteobacteria) and unclassified PLTA13 (Gammaproteobacteria), indicating that multiple classes within this phylum may exhibit sensitivity to the introduced consortium. Both unclassified A21b and unclassified PLTA13 (Gammaproteobacteria) represent lineages described solely based on environmental 16S rRNA sequences and lack cultivated representatives; consequently, their ecology and physiology remain largely unknown despite their frequent detection in soil and rhizosphere surveys [43,44,45]. As a result, the ecological relevance of the observed reductions in their relative abundance following PP consortium application remains difficult to assess.

At the phylum level, a decrease was observed only for Thermodesulfobacteriota, and this effect did not persist over time. In the present study, the relative abundance of Thermodesulfobacteriota remained below 0.3%, suggesting that these taxa likely do not play a major functional role in the studied soil ecosystem. Moreover, members of this phylum are predominately described as thermophilic, anaerobic sulfate-reducing bacteria associated with high-temperature or sulfur-rich environments, such as hot springs and anoxic sediments, rather than as typical constituents of temperate agricultural soils [46,47]. Consequently, the ecological relevance of their sporadic detection in soil microbiome surveys remains unclear.

As noted above, information on the effects of PGPB on the native bacterial community associated with barley remains scarce. Nevertheless, previous studies employing the commercial preparation Proradix^®^ (containing *Pseudomonas* sp. DSMZ 13134) reported transient changes in the structure of the dominant root-associated bacterial community of barley that persisted for up to three weeks post-application [20]. However, those observations were based on a T-RFLP approach that provides substantially lower taxonomic resolution than contemporary high-throughput sequencing methods [41]. Other studies have not assessed taxonomic composition but focused on the effects of PGPB on physiological groups of soil microorganisms using classical approaches or on overall soil microbial activity [15,36]. For instance, Niewiadomska et al. [15] demonstrated that the application of a bacterial consortium composed of *Bacillus subtilis*, *B. amyloliquefaciens*, and *P. fluorescens* increased soil dehydrogenase activity relative to untreated control soils.

## 5. Conclusions

The results of this field study demonstrate that inoculation of spring barley with a bacterial consortium composed of *Paenibacillus* sp. Z15 and *Pseudomonas* sp. KR227 can effectively enhance plant performance under production conditions. Importantly, application of the consortium did not induce major alterations in the structure or diversity of the native rhizosphere bacterial community, as revealed by high-resolution 16S rRNA gene sequencing. The limited and transient shifts observed in the relative abundance of selected microbial taxa suggest that the introduced PGPB strains can support plant growth without causing long-term ecological disturbances to the soil microbiome. This microbiome-neutral behavior is particularly relevant in the context of sustainable agriculture, as it indicates that yield improvements can be achieved without compromising microbial stability within the soil ecosystem.

At the same time, it should be acknowledged that the proposed growth-promoting mechanisms, including IAA production, phosphate solubilization, and nitrogen fixation, were inferred on the basis of strain identity and previously reported functional traits rather than through direct functional measurements conducted under field conditions. Therefore, future studies should aim to more explicitly characterize the underlying mechanisms of plant growth promotion, including the quantification of IAA and nitrogen and phosphorus contents in plant tissues at different developmental stages along the BBCH scale.

Furthermore, it remains to be determined whether the subtle taxonomic shifts within the native rhizobacterial communities translate into functional changes in the indigenous microbiota. This could be addressed in future research through the integration of metagenomic, metatranscriptomic, or metabolomic approaches. In addition, the persistence of the bacterial consortium in the rhizosphere could be monitored using qPCR. Finally, long-term and multi-site field experiments are required to evaluate the stability and reproducibility of the consortium’s effects across different soil types, climatic conditions, and barley cultivars.

## Figures and Tables

**Table 1:** Physico-chemical properties of the barley rhizosphere.

Treatment	TOC [%]	pH (KCl)	TKN [%]	TKN [mg/kg]	N-NH_4_ [mg/kg]	N-NO_3_ [mg/kg]	AP [mg/kg]	TP [mg/kg]
Term I								
Control	0.95 ± 0.22 a	6.20 ± 0.1 a	0.11 ± 0 a	1097 ± 31 a	5.60 ± 1.59 a	3.57 ± 0.72 a	277.33 ± 6.11 a	629.33 ± 33.62 a
PP	0.98 ± 0.16 a	6.17 ± 0.12 a	0.11 ± 0.01 a	1056 ± 61 a	5.40 ± 0.35 a	2.25 ± 1.21 a	276.33 ± 20.11 a	616.33 ± 24.13 a
Term II								
Control	0.88 ± 0.11 a	6.83 ± 0.12 a	0.11 ± 0.03 a	1058 ± 285 a	5.67 ± 0.31 a	3.23 ± 0.51 a	224.00 ± 5.29 a	559.33 ± 61.92 a
PP	0.78 ± 0.04 a	7.00 ± 0.1 a	0.12 ± 0.02 a	1148 ± 171 a	5.47 ± 0.12 a	4.73 ± 1.64 a	229.33 ± 10.02 a	529.00 ± 3.61 a

PP—inoculation with bacterial consortium. Different letters in the same column in the same sampling term indicate significant differences between means at *p* < 0.05 according to Tukey’s HSD test.

**Table 2:** Biometric and yield parameters of barley.

Treatment	Shoot Height [cm]	Shoot Yield [g/m^2^]	Grain Yield [g/m^2^]	Root Mass I Term [g]	Root Mass II Term [g]	TGW [g]
Control	72.38 ± 2.41 b	1049 ± 53 a	515 ± 41 b	2.74 ± 0.93 b	8.36 ± 0.93 a	4.79 ± 0.37 a
PP	78.01 ± 1.45 a	1234 ± 189 a	595 ± 43 a	6.03 ± 1.00 a	13.41 ± 3.72 a	4.74 ± 0.24 a

PP—inoculation with bacterial consortium. Different letters in the same column indicate significant differences between means at *p* < 0.05 according to Tukey’s HSD test.

**Table 3:** Alpha diversity.

Sample Name	Chao1	Observed Features	Shannon	Simpson
Term I				
Control	2484 ± 441 a	2457 ± 445 a	10.34 ± 0.24 a	0.998 ± 0.000 a
PP	2510 ± 213 a	2485 ± 211 a	10.33 ± 0.22 a	0.998 ± 0.001 a
Term II				
Control	1799 ± 193 a	1778± 182	9.88 ± 0.08 a	0.998 ± 0.000 a
PP	1946± 167 a	1926± 167 a	9.94 ± 0.13 a	0.998 ± 0.001 a

PP—inoculation with bacterial consortium. Control and PP—term I; Control2 and PP2—term II. Different letters indicate significant differences between means at *p* < 0.05 according to Tukey’s HSD test.

## Data Availability

All raw illumina sequencing data generated in this study have been deposited in the NCBI Sequence Read Archive (SRA) under the BioProject accession number PRJNA1403289.

## Supplementary Materials

The following supporting information can be downloaded at https://www.mdpi.com/article/10.3390/microorganisms14020488/s1, Figure S1: Phylogenetic tree based on concatenated 16S rRNA and *gyrB* sequences showing the relationship of strain Z15 to selected *Paenibacillus* strains. The tree was constructed using the Maximum Likelihood method (Tamura–Nei model, 1000 bootstrap replicates) in MEGA 12; Figure S2: Barplot of PICRUSt function annotation; a—first time point, b—second time point

## References

[1] Food and Agriculture Organization of the United Nations (FAO) (2023). Production: Crops and Livestock Products.

[2] Baik B.K., Ullrich S.E. (2008). Barley for food: Characteristics, improvement, and renewed interest. J. Cereal Sci..

[3] Tyagi J., Ahmad S., Malik M. (2022). Nitrogenous fertilizers: Impact on environment sustainability, mitigation strategies, and challenges. Int. J. Environ. Sci. Technol..

[4] Amin F., Jilani M.I. (2024). Environmental, Microbiological and Chemical Implications of Fertilizers use in soils: A review. Int. J. Chem. Biochem. Sci..

[5] Heuser I. (2022). Soil governance in current European Union law and in the European Green Deal. Soil Secur..

[6] Dobrzyński J., Kulkova I., Jakubowska Z., Naziębło A., Wróbel B. (2024). *Pseudomonas* sp. G31 and *Azotobacter* sp. PBC2 Changed Structure of Bacterial Community and Modestly Promoted Growth of Oilseed Rape. Int. J. Mol. Sci..

[7] Dobrzyński J., Kulkova I. (2025). *Paenibacillus peoriae*: Current knowledge and agricultural biotechnology potential of a close relative of *P. polymyxa*. Antonie Van Leeuwenhoek.

[8] Olanrewaju O.S., Glick B.R., Babalola O.O. (2017). Mechanisms of action of plant growth promoting bacteria. World J. Microbiol. Biotechnol..

[9] Maciel-Rodríguez M., Moreno-Valencia F.D., Plascencia-Espinosa M. (2025). The role of plant growth-promoting bacteria in soil restoration: A strategy to promote agricultural sustainability. Microorganisms.

[10] Dobrzyński J., Jakubowska Z. (2025). *Pseudomonas protegens* as a biocontrol agent against phytopathogenic fungi—A mini review. World J. Microbiol. Biotechnol..

[11] Naziębło A., Pytlak A., Furtak A., Dobrzyński J. (2026). Advances and hotspots in research on Verrucomicrobiota: Focus on agroecosystems. Microb. Ecol..

[12] Ngalimat M.S., Mohd Hata E., Zulperi D., Ismail S.I., Ismail M.R., Mohd Zainudin N.A.I., Saidi N.B., Yusof M.T. (2021). Plant growth-promoting bacteria as an emerging tool to manage bacterial rice pathogens. Microorganisms.

[13] Naziębło A., Dobrzyński J. (2025). Biotransformation of As, Cr, Hg, and Mn by Pseudomonadota: Chances and risks. Biodegradation.

[14] Jakubowska Z., Gradowski M., Dobrzyński J. (2025). Role of plant growth-promoting bacteria (PGPB) in enhancing phenolic compounds biosynthesis and its relevance to abiotic stress tolerance in plants: A review. Antonie Van Leeuwenhoek.

[15] Niewiadomska A., Płaza A., Wolna-Maruwka A., Budka A., Głuchowska K., Rudziński R., Kaczmarek T. (2023). Consortia of plant growth-promoting rhizobacteria and selected catch crops for increasing microbial activity in soil under spring barley grown as an organic farming system. Appl. Sci..

[16] Zaib S., Zubair A., Abbas S., Hussain J., Ahmad I., Shakeel S.N. (2023). Plant growth-promoting rhizobacteria (PGPR) reduce adverse effects of salinity and drought stresses by regulating nutritional profile of barley. Appl. Environ. Soil Sci..

[17] Dolkhani F., Bijanzadeh E., Boostani H.R., Hardie A.G. (2022). Effect of nitrogen-fixing bacteria application on biochemical properties, yield, and nutrients of barley. J. Soil Sci. Plant Nutr..

[18] Kaur T., Devi R., Kumar S., Sheikh I., Kour D., Yadav A.N. (2022). Microbial consortium with nitrogen fixing and mineral solubilizing attributes for growth of barley (*Hordeum vulgare* L.). Heliyon.

[19] Kouas S., Djedidi S., Debez I.B.S., Sbissi I., Alyami N.M., Hirsch A.M. (2024). Halotolerant phosphate solubilizing bacteria isolated from arid area in Tunisia improve P status and photosynthetic activity of cultivated barley under P shortage. Heliyon.

[20] Buddrus-Schiemann K., Schmid M., Schreiner K., Welzl G., Hartmann A. (2010). Root colonization by *Pseudomonas* sp. DSMZ 13134 and impact on the indigenous rhizosphere bacterial community of barley. Microb. Ecol..

[21] Dobrzyński J., Kulkova I., Jakubowska Z., Wróbel B. (2024). Non-Native PGPB Consortium Altered the Rhizobacterial Community and Slightly Stimulated the Growth of Winter Oilseed Rape (*Brassica napus* L.) under Field Conditions. Microb. Ecol..

[22] Dobrzyński J., Naziębło A., Kulkova I., Szpytma M., Antosik A., Jakubowska Z., Wróbel B. (2026). Response of triticale and its native rhizobacterial community to inoculation with a consortium of *Paenibacillus* sp. Z15 and *Pseudomonas* sp. KR227. World J. Microbiol. Biotechnol..

[23] Yamamoto S., Harayama S. (1995). PCR amplification and direct sequencing of *gyrB* genes with universal primers and their application to the detection and taxonomic analysis of *Pseudomonas putida* strains. Appl. Environ. Microbiol..

[24] Dobrzyński J., Kulkova I., Jakubowska Z., Wróbel B. (2025). Non-native PGPB consortium consisting of *Pseudomonas* sp. G31 and *Azotobacter* sp. PBC2 promoted winter wheat growth and slightly altered the native bacterial community. Sci. Rep..

[25] Mendes L.W., Raaijmakers J.M., De Hollander M., Mendes R., Tsai S.M. (2018). Influence of resistance breeding in common bean on rhizosphere microbiome composition and function. ISME J..

[26] (2022). Soil, Sludge and Treated Biowaste—Determination of pH.

[27] (2022). Sludge, Treated Biowaste and Soil—Determination of Loss on Ignition—Dry Matter—Organic Matter.

[28] (2012). Sludge, Treated Biowaste and Soil—Determination of Kjeldahl Nitrogen.

[29] (2021). Soil, Treated Bio-Waste, Sewage Sludge and Waste—De-Treatment of Fractions of Royal Water-Soluble Elements.

[30] (2024). Environmental Solid Matrices—Determination of Elements Using Inductively Coupled Plasma Optical Emission Spectrometry (ICP-OES).

[31] Arnon D.I. (1949). Copper enzymes in isolated chloroplasts. Polyphenoloxidase in *Beta vulgaris*. Plant Physiol..

[32] Azeem M., Haider M.Z., Javed S., Saleem M.H., Alatawi A. (2022). Drought stress amelioration in maize (*Zea mays* L.) by inoculation of *Bacillus* spp. strains under sterile soil conditions. Agriculture.

[33] STATSOFT, Inc. (2014). STATISTICA (Data Analysis Software System), Version 13.1.

[34] Yahya M., Islam E.U., Rasul M., Farooq I., Mahreen N., Tawab A., Irfan M., Rajput L., Amin I., Yasmin S. (2021). Differential root exudation and architecture for improved growth of wheat mediated by phosphate solubilizing bacteria. Front. Microbiol..

[35] Wang Z., Zhang H., Liu L., Li S., Xie J., Xue X., Jiang Y. (2022). Screening of phosphate-solubilizing bacteria and their abilities of phosphorus solubilization and wheat growth promotion. BMC Microbiol..

[36] Hosseini E., Zarei M., Sepehri M., Safarzadeh S. (2021). Do bagasse biochar and microbial inoculants positively affect barley grain yield and nutrients, and microbial activity?. J. Plant Nutr..

[37] Jiménez-Gómez A., Saati-Santamaría Z., Kostovcik M., Rivas R., Velázquez E., Mateos P.F., Menéndez E., García-Fraile P. (2020). Selection of the root endophyte *Pseudomonas brassicacearum* CDVBN10 as plant growth promoter for *Brassica napus* L. crops. Agronomy.

[38] Chen Y., Li S., Liu N., He H., Cao X., Lv C., Dai J. (2021). Effects of different types of microbial inoculants on available nitrogen and phosphorus, soil microbial community, and wheat growth in high-P soil. Environ. Sci. Pollut. Res..

[39] Wolińska A., Kuźniar A., Zielenkiewicz U., Izak D., Szafranek-Nakonieczna A., Banach A., Błaszczyk M. (2017). Bacteroidetes as a sensitive biological indicator of agricultural soil usage revealed by a culture-independent approach. Appl. Soil Ecol..

[40] Kruczyńska A., Kuźniar A., Podlewski J., Słomczewski A., Grządziel J., Marzec-Grządziel A., Wolińska A. (2023). Bacteroidota structure in the face of varying agricultural practices as an important indicator of soil quality—A culture-independent approach. Agric. Ecosyst. Environ..

[41] Cassán F., Diaz-Zorita M. (2016). Azospirillum sp. in current agriculture: From the laboratory to the field. Soil Biol. Biochem..

[42] Cassán F., Coniglio A., López G., Molina R., Nievas S., de Carlan C.L.N., Donadio F., Torres D., Rosas S., Pedrosa F.O. (2020). Everything you must know about *Azospirillum* and its impact on agriculture and beyond. Biol. Fertil. Soils.

[43] Spain A.M., Krumholz L.R., Elshahed M.S. (2009). Abundance, composition, diversity and novelty of soil Proteobacteria. ISME J..

[44] Sjöberg S., Stairs C.W., Allard B., Homa F., Martin T., Sjöberg V., Dupraz C. (2020). Microbiomes in a manganese oxide producing ecosystem in the Ytterby mine, Sweden: Impact on metal mobility. FEMS Microbiol. Ecol..

[45] Besze B.Z., Borsodi A.K., Megyes M., Zsigmond T., Horel Á. (2024). Changes in the taxonomic composition of soil bacterial communities under different inter-row tillage managements in a sloping vineyard of the Balaton Uplands (Hungary). Biol. Futur..

[46] Bhatnagar S., Badger J.H., Madupu R., Khouri H.M., O’Connor E.M., Robb F.T., Ward N.L., Eisen J.A. (2015). Genome sequence of a sulfate-reducing thermophilic bacterium, *Thermodesulfobacterium commune* DSM 2178T (phylum Thermodesulfobacteria). Genome Announc..

[47] Mardanov A.V., Beletsky A.V., Kadnikov V.V., Slobodkin A.I., Ravin N.V. (2016). Genome analysis of *Thermosulfurimonas dismutans*, the first thermophilic sulfur-disproportionating bacterium of the phylum Thermodesulfobacteria. Front. Microbiol..

