# Supplementary material for: Paenibacillus–Pseudomonas Consortium Improves Barley Performance with Minimal Impact on Native Rhizobacterial Community
Source: Microorganisms. 2026 Feb 18;14(2):488. doi: 10.3390/microorganisms14020488 (PMC12943435; doi:10.3390/microorganisms14020488)
Supplement: Supplementary file 1 [file microorganisms-14-00488-s001.zip › microorganisms-4127177-supplementary.pdf]

Paenibacillus–Pseudomonas consortium improves barley performance with minimal impact on native rhizobacterial community

Jakub Dobrzyński[1]*, Aleksandra Naziębło[1], Iryna Kulkova[1], Magdalena Szpytma[1], Adrianna Antosik[2], Monika Sitarek-Andrzejczyk[1], and Barbara Wróbel[1]

[1]Institute of Technology and Life Sciences-National Research Institute, Falenty, 3 Hrabska Avenue, 05-090, Raszyn, Poland
[2]Ekosystem-Nature's Heritage Association, Institute of Microbial Technologies, Al. NSZZ Solidarność 9, 62-700 Turek, Poland

*Correspondence: j.dobrzynski@itp.edu.pl

>*gyrB* Z15

GNGNANCCANAANGNCNNGGGGGGTCGGNNNNGNGCNANCGTGCACTCTGTATAGAACGCTCTTTCGGAATGGCTT
GAAGTAGAAATTTACCGGGACGGCAAGATTCACCGTCAGCGGTTTGAATATTGGCAGGACAAGAAGGGCGTGGAGCA
ATGTTCGAGGAACCGGACCCACAGGCCTTGGAAGTGCTGGGCAATNACTAACAAGACGGGCTCGAAAAATTACATTTT
AAACCCGGATATTTCGTGTTTTTCAGGCAGGCATTCATTTTAANCTACGATACGTTGGCTGAGCGCCCTTCAGGAAAAT
TGCTTTTTTCTAAATTTCGGGCCTTCGTATTTCAACTTAAAGACGAACGCAGCGGAAAGTCAGATGAGTATTTTTATGAG
GGTGGCGCAAGTCAGTTTGTTGCTTTTCTGAATGAGGGCAAGGATGTGCTGCATGACGTTATTCACTTTAATGCCGAGA
AAGAAGACATTGAAGTAGAGATTGCCATCCAGTACAATGCGGGTTATACAGAGACGATTGCTTCGTTCGTTAACTCCAT
TCCGACACGTGGCGGAGGTACGCATGAAACGGGATTCAAAACCGCTTACACTCGTGTCATGAACGACTATGCGCGCAA
AACGGTGATGTTGAAAAGAAAAGGATAAAAACTTGGAGGGCAACGATCTACGTGAGGGCATGATGGCTGTAATCAGT
GTCAAAATGGCTGAAGTTGAATTTGTCGGCCAGACAAAGGATCAGCTGGGTAGCGCTTCGGCACGGAGTACAGTGGAT
GCCATCGTATCTGAGCAGATGCAGCGTTTTTTGGAAGAAAATCCGCAGATAGCACAAACTTTGATCAAGAAGGCAGTT
CAAGCATCCAGAGCGCGTGAAGCTGCACGTAAAGCTCGGGATGAGATGCGTTCCNGGTAAAAAAGCGCAGTGAAAGT
TTCCAATTTGAATGGTAAACTGGTCGCCTGCNGCAGTCCAAGGGATTTTTACACGTAATGAGTTGTTTATTGTGGAAGG
CGNATTCGGCTGGGAGGAATCAGCCAAGCAGGGACCGGGATTTTCCAAAATTCAGGCCATATTGCCGCTAAAGGGCA
AGCCGATGAATCCGGAAAAATCCAAACTGGCGGATATTATGAAGAATGATGAGTACCGTGCTATTACAGCAGCTATTG
TGCGGNTTTGGAACACACTTCCCCTTGCCCAGCAAGANCGATGACACCTACTAG

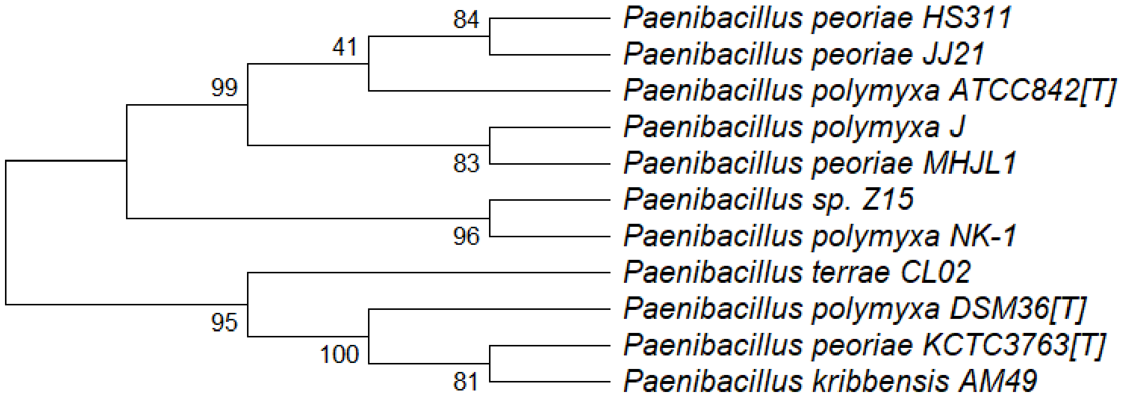

Figure S1. Sequence of *gyrB* and phylogenetic tree based on concatenated 16S rRNA and *gyrB* sequences showing the relationship of strain Z15 to selected *Paenibacillus* strains. The tree was constructed using the Maximum Likelihood method (Tamura–Nei model, 1000 bootstrap replicates) in MEGA 12.

a)

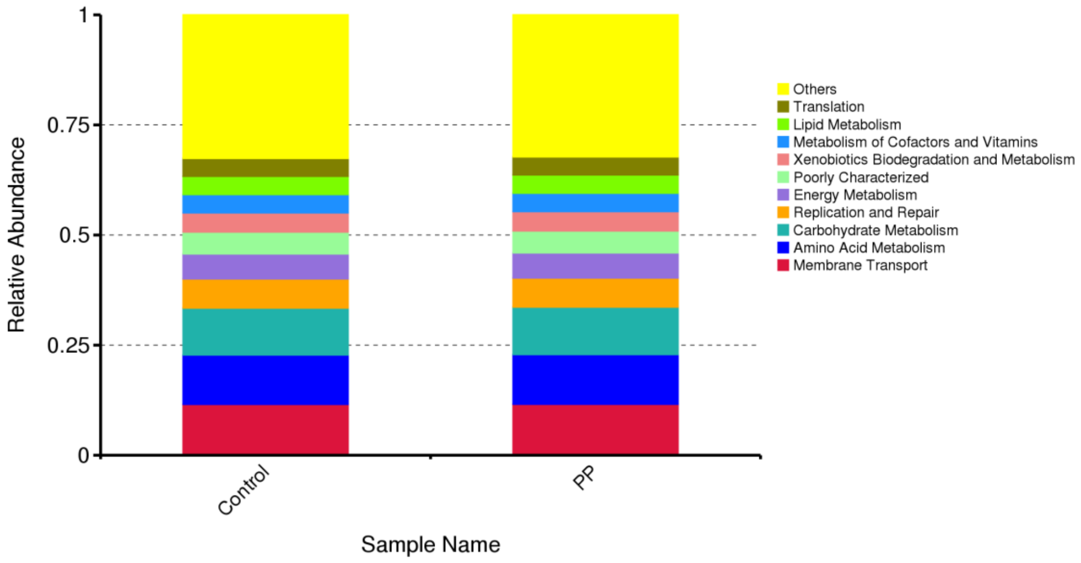

b)

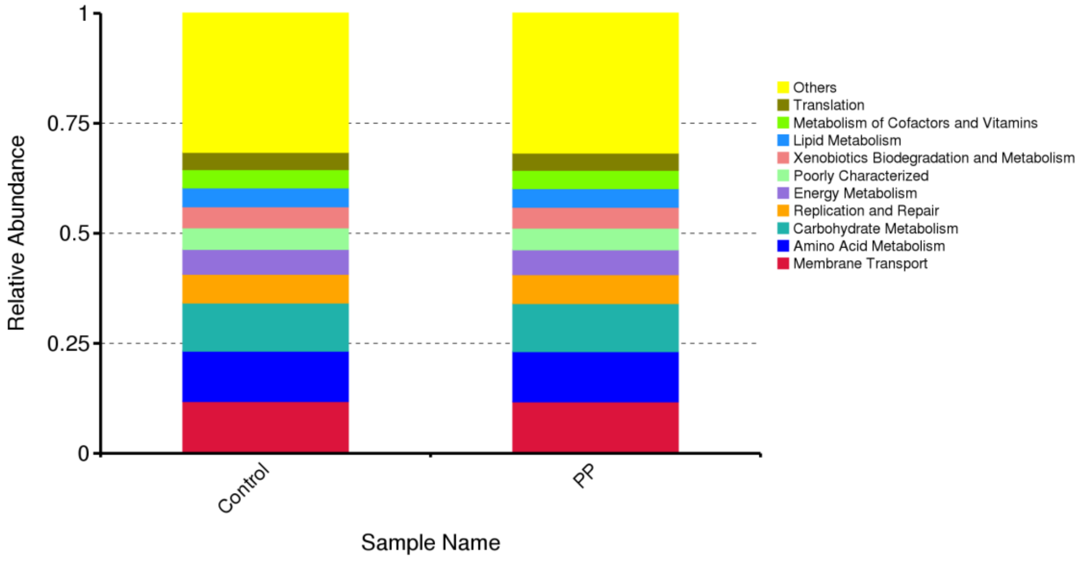

Figure S2. Barplot of PICRUSt function annotation; a – first time point, b – second time point
